# *Neisseria meningitidis* Sequence Type and Risk for Death, Iceland

**DOI:** 10.3201/eid1207.051624

**Published:** 2006-07

**Authors:** Magnús Gottfreðsson, Mathew A. Diggle, David I. Lawrie, Helga Erlendsdóttir, Hjördís Harðardóttir, Karl G. Kristinsson, Stuart C. Clarke

**Affiliations:** * Landspítali University Hospital, Reykjavík, Iceland;; † University of Iceland, Reykjavík, Iceland;; ‡ Scottish Meningococcus and Pneumococcus Reference Laboratory, Glasgow, United Kingdom;; § University of Glasgow, Glasgow, United Kingdom

**Keywords:** Neisseria meningitidis, multilocus sequence typing (MLST), meningococcal disease, meningitis, epidemiology, mortality

## Abstract

Invasive meningococcal infections are hyperendemic in Iceland, a relatively isolated country in the mid-Atlantic. We performed a nationwide study on all viable meningococcal strains (N = 362) from 1977 to 2004. We analyzed the association of patient's age and sex, meningococcal serogroups, and sequence types (STs) with outcomes. Overall, 59 different STs were identified, 19 of which were unique to Iceland. The most common STs were 32 (24.6%), 11 (19.9%), and 10 (10.2%). The unique ST-3492 ranked fourth (7.7%). The most common serogroups were B (56.4%), C (39.8%), and A (2.2%). Age (p<0.001) and infection with a unique ST (p = 0.011) were independently associated with increased death rates, whereas isolation of meningococci from cerebrospinal fluid only was associated with lower death rates (p = 0.046). This study shows evolutionary trends of meningococcal isolates in a relatively isolated community and highlights an association between unique STs and poor outcome.

Invasive infections caused by *Neisseria meningitidis* (meningococci) cause high rates of illness and death worldwide (*1**–**3*). Meningococci have frequently caused epidemics in Iceland, a relatively isolated community in the mid-Atlantic (*4**,**5*). To more fully understand the phylogeny of meningococcal strains, various typing methods have been used, including serogroup and serotype classifications. Epidemiologic studies have used more discriminating methods, such as multilocus enzyme electrophoresis, based on electrophoretic variation of several chromosomally encoded cytoplasmic "housekeeping" enzymes (*6*). More recently, sequence-based molecular methods have been used to type meningococci. Multilocus sequence typing (MLST) uses neutrally selected housekeeping genes (*7*), which are sequenced with automated equipment (*8*). This method gives all the information obtained by multilocus enzyme electrophoresis and improves on it in several ways (*7*). MLST is not dependent on the researcher's interpretation, and no reference standards are necessary. The data are portable; they are easily stored and transmitted and can therefore be easily compared.

We have generated a population-based registry of invasive meningococcal infections in Iceland since 1975. Iceland is well suited for studies of meningococcal infections, since the population is well defined, patient follow-up information is relatively accessible, and meningococcal isolates dating back to 1977 are stored centrally. We used MLST to study the evolutionary dynamics of invasive meningococcal infections in Iceland during a 28-year period, 1977–2004. The purpose of this long-term, nationwide study was 2-fold: 1) compare Icelandic strains with those circulating globally and 2) study the association between patient demographics, sequence types (STs), serogroups, and outcomes.

## Materials and Methods

### Setting

Iceland is a 103,000-km^2^ island in the mid-Atlantic, with a population of 220,918 at the beginning of the study period and 293,577 at the end of 2004. Every citizen has access to government-based health care. Currently, 2 university hospitals and 14 community hospitals exist in the country. Since 1975, blood cultures for the whole country have been processed at only 3 sites. This study was approved by the National Bioethics Committee of Iceland and the Data Protection Authority of Iceland.

### Case Definitions and Collection of Data

A prospective registry of all invasive cases of meningococcal disease since 1975 has been generated. This registry includes all patients with a diagnosis of infection, confirmed by culture of blood, cerebrospinal fluid (CSF), or joint fluid. It also includes patients with clinical illness compatible with meningococcal disease and a positive culture from a throat specimen or a positive Gram-stain smear, latex agglutination test, or polymerase chain reaction (PCR) of CSF, blood, or joint fluid. The registry also includes information regarding patient age, sex, and residence and location of hospital where treatment was administered. We calculated the death ratio for patients with meningococcal disease during hospitalization or within 4 weeks of diagnosis by hospital records and the national population registry of Iceland (http://www.statice.is/). Imported cases were excluded.

### Microbiology

All invasive meningococcal isolates are sent for serogrouping and susceptibility testing at the Department of Clinical Microbiology at Landspitali University Hospital, the national reference laboratory for the country. The oldest invasive isolates in the collection date from 1977. In total, 362 isolates from January 1, 1977, to December 31, 2004, were viable and thus available for further study. Serogrouping was performed by using standard antisera (Difco Laboratories, Detroit, MI, USA). When an unusual relationship was observed between serogroups and STs, serogrouping was performed at least twice. MICs for penicillin, sulfadiazine, and rifampin were measured by using the Etest (AB Biodisk, Solna, Sweden) according to Clinical Laboratory Standards Institute criteria (*9*).

### MLST

MLST was performed by determining the nucleotide sequences of 7 housekeeping genes (*abcZ*, *adk*, *aroE*, *fumC*, *gdh*, *pdhC*, *pgm*) as previously described (*8*). Alleles and sequences were assigned by using the MLST database (http://neisseria.org/nm/typing/mlst/). Sequence typing data were analyzed as described previously (*10*). Data were submitted to the MLST database from January 30, 2004, to August 10, 2004, and STs that had not been previously described were assigned a new number. Strains with STs that were found exclusively in Iceland were classified as "unique" in the context of statistical analysis. The allelic profiles were used to study the relatedness of the STs by using the unweighted pair-group method with arithmetic mean (UPGMA). Phylogenetic trees were constructed with the Sequence Type Analysis and Recombinational Tests (START) suite of programs (http://pubmlst.org/software/).

BURST (Based Upon Related Sequence Types, http://pubmlst.org/software/) was also used to examine the relationships within clonal complexes, while the relationships between different clonal complexes were ignored. BURST required allelic profile data only, and these also contained their ST numbers. For MLST data based on 7 loci, a cutoff point of 5 identical loci allows inclusion of strains that belong to a single clonal complex, while excluding those that do not.

### Statistical Analysis

The collective term "ST group" was used to differentiate unique STs, defined as strains found exclusively in Iceland, from other STs, which have been described elsewhere. We used the Pearson χ^2^ test and the Fisher exact test as appropriate to assess the bivariate relationship between categorical variables, in particular how death rate was related to the other variables, including ST group. Patient age, based on ST group of the isolate and patient status, was compared by using the Mann-Whitney test. To further assess factors associated with death, we performed multivariable logistic regression analysis with death as the dependent variable. Controlling for age, we tested each of the following variables in separate models: sex of the patients, serogroup (B, C, and others), ST group (unique STs vs. other STs), residence (capitol area vs. rural), hospital location (capitol area vs. rural), and finally, we examined the site of the positive bacterial culture in 2 different ways, in 4 categories (blood, CSF only, both blood and CSF, and other sites), and in 2 categories (CSF only vs. all other sites). Variables that remained significant in the model after controlling for age were evaluated in further models to assess independent associations with death. Level of significance was set at p<0.05. All tests were 2-tailed. Statistical analysis was performed by using SPSS version 10.5 (SPSS Inc., Chicago, IL, USA).

## Results

### Epidemiology of Invasive Meningococcal Disease

**Figure 1:**
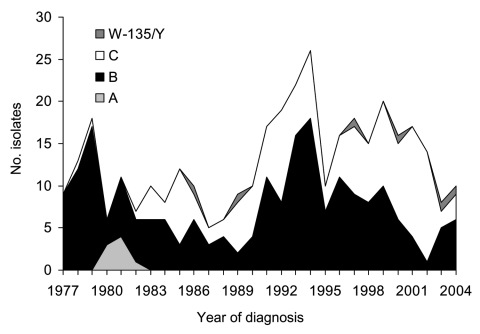
Annual number of invasive meningococcal isolates by serogroup, Iceland, 1977–2004.

The number of registered cases in Iceland varied greatly from 1977 to 2004, ranging from 55 cases/year during the epidemic of 1977 to 7–8 cases/year in 1988 and 2003. The average incidence of invasive meningococcal disease during this 28-year period was 7.1 cases/100,000 population/year, but if the epidemic year of 1977 is excluded, it drops to 6.4 cases/100,000 population/year. A detailed description of the study cohort and serogroups of the organisms is given in Table 1. Meningococci were most commonly isolated from CSF only (39.7%). Serogroups varied substantially within the study period (Figure 1).

**Table 1:** Description of the patient cohort

Parameter	No. (%)
Patients	362
Male	185 (51.1)
Female	177 (48.9)
Children*	244 (67.4)
Adults	118 (32.6)
Strain isolated from cerebrospinal fluid only	144 (39.8)
Strain isolated from blood only	105 (29.0)
Strain isolated from cerebrospinal fluid and blood	78 (21.5)
Strain isolated from joint fluid	10 (2.8)
Strain grown from throat culture†	25 (6.9)
Serogroup A	8 (2.2)
Serogroup B	204 (56.4)
Serogroup C	144 (39.8)
Serogroup Y	3 (0.8)
Serogroup W135	3 (0.8)

*<16 years of age at the time of diagnosis. †Positive throat culture in the setting of invasive meningococcal disease, diagnosed clinically.

### Sequence Typing of Meningococcal Isolates

MLST was performed on all 362 viable strains, which were responsible for 72.7% of all documented cases of invasive meningococcal disease in the country during the study period. Overall, 59 STs were observed. A summary of the MLST results is given in Table 2, and the association between STs and serogroups is shown in Table 3. Missing isolates were predominantly from the first 2 years of the study. During the epidemic of 1977, pathogens were genetically homogenous, however, as all strains were ST-10 (Table 2).

**Table 2:** Epidemiology of invasive meningococcal disease in Iceland *

Year (no. cases)	No. isolates	Sequence type (ST)																			
		1	8	10	11	32	40	41	44	60	162	206	275	1314	1323	*2148*	2266	*3435*	*3464*	*3492*	Other STs
1977 (55)	9			9																	
1978 (21)	13			10																	334, 2986, *3333*
1979 (25)	18			13		1												2			13, 1011
1980 (16)	6	3		1																	*3501*, *4013*
1981 (18)	11	4		2		2															1423, 2320, *3471*
1982 (10)	7	1		1		2								1	1			1			
1983 (19)	10			1		2							1	2	2					1	43
1984 (13)	8					6									1				1		
1985 (13)	12									2		1			3				1	3	1328 (×2)
1986 (11)	10					1				2		1			2					3	23
1987 (9)	5											1	2	1					1		
1988 (7)	6					1				2				1						2	
1989 (14)	9				1									1						5	785, *3502*
1990 (17)	10					2						1		1						4	2843, *3508*
1991 (18)	17				1	5					1	2		1						3	1015, 1154, *3509*, *5119*
1992 (21)	19				4	4						3	1	1						3	34, *3710* (×2)
1993 (23)	22				5	13		2	1											1	
1994 (30)	26		1		5	16								1						1	286, *3756*
1995 (14)	10				2	5			1											1	46
1996 (17)	16		1		3	8								1						1	43,467
1997 (20)	18		6		2	5			1	1											22, 944, 1671
1998 (16)	15		4		3	7											1				
1999 (20)	20		1		10	4		1								1					352, *3757*, *3758*
2000 (18)	16		3		6	2					1					1					23,34*,3759*
2001 (19)	17				12	1			1							1					33, 1943
2002 (16)	14				13												1				
2003 (8)	8				2						1						3				1163, *3334*
2004 (10)	10				3	2	3														1281, *4178*
Total	362	8	16	37	72	89	3	3	4	7	3	9	4	11	9	3	5	3	3	28	45

*The total number of cases is shown with the number of ST isolates available for multilocus sequence typing. Unique STs, those found exclusively in Iceland , are shown in *italics*. The most prevalent STs for each year are shown in shaded cells.

**Table 3:** Association between meningococcal sequence types (STs), serogroups, and death, Iceland, 1977–2004*

ST	Serogroup A		Serogroup B		Serogroup C		Total	
	No. isolates	No. deaths	No. isolates	No. deaths	No. isolates	No. deaths	No. isolates	No. deaths (%)
32			86	7	3		89	7 (8)
11			5		67	6	72	6 (8)
10			36	1	1	1	37	2 (5)
3492			2		26	4	28	4 (14)
8					16		16	
1314			11				11	
206			1	1	8		9	1 (11)
1323					9		9	
1	8						8	
60			7				7	
2266			5				5	
44			4				4	
275			4	1			4	1 (25)
40			3				3	
41			3	1			3	1 (33)
162			3				3	
2148			2	1	1		3	1 (33)
3435			3	2			3	2 (67)
3464					3		3	
23							2†	1 (50)
34			1		1		2	
43			1		1		2	
1328					2	1	2	1 (50)
3710			2				2	

*All STs that were encountered more than once are shown. Blank cells indicate zero values. Unique STs are shown in italics; 19 unique STs were found. Of 362 cases, 53 were caused by strains with unique STs and 309 by other STs. †Both isolates were serogroup Y.

Strains of 8 different STs caused 75% of all infections. ST-32 was most common, causing 24.6% of all cases. It was predominantly of serogroup B and endemic during almost the entire period. ST-32 also caused a small epidemic in the country in 1993 and 1994. The second most common ST was ST-11 (19.9% of all cases), which was predominantly of serogroup C. It was first seen in Iceland in 1989 and was the main culprit in invasive meningococcal disease from 1999 to 2002. ST-10 caused 10.2% of all infections and dominated during the first 3 years of the study, but it has not been seen since 1983. In 1983, a new type emerged, ST-3492 from the ST-41/44 complex; it was the fourth most common ST in Iceland overall and caused 7.7% of all infections. ST-3492 was predominantly serogroup C. This ST was the most common cause of invasive disease in 1989 and 1990 but disappeared after 1996.

During the study, 19 STs that were unique to Iceland were described, and these accounted for 14.6% of all invasive infections. Most of these emerging STs (14 of 19) caused only single infections (3.9% of all episodes). The remaining 5 STs caused 10.8% of all invasive disease in the country.

In general, good concordance was seen between STs and serogroups. Nevertheless, isolates exhibiting both serogroup B and C capsules were observed among the 4 most common STs (Table 3).

### Dendrogram and Clonal Complexes

**Figure 2:**
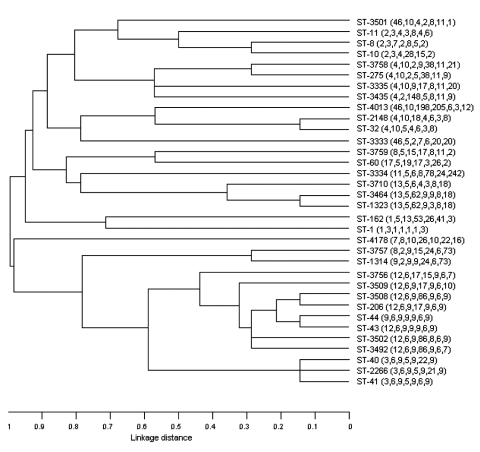
Phylogenetic relationships of invasive meningococcal isolates in Iceland, 1977–2004. Unweighted pair-group method with arithmetic mean (UPGMA) was used to construct the tree with the Sequence Type Analysis and Recombinational Tests (START) suite of programs. Sequence types (STs) and allelic profiles are given on the right. All STs that were encountered in >3 clinical cases and all new STs are shown.

The phylogenetic tree of isolates in this study is shown in Figure 2. All STs that were encountered in >3 clinical cases and all new STs are shown. ST-2148 was remarkably similar to ST-32, differing only at 1 locus. This clone emerged in 1999 and, like ST-32, was found in both serogroups B and C. ST-11 was most closely related to ST-8 and ST-10, which had 4 and 3 genes, respectively, in common with ST-11. However the ST-8 and ST-10 clones had different serogroups. The relationships within clonal complexes and their association with death is shown in Table 4. We identified 9 complexes and 17 singletons; most isolates (26.5%) fell within group 2, in which ST-32 was the ancestral strain.

**Table 4:** Association between *Neisseria meningitidis* CC, ST, and patient deaths, Iceland, 1977–2004*†

CC	ST	Deaths (freq)	SLV	DLV	SAT
1	10	2 (37)	0	1	0
	8	0 (16)	0	1	0
2	**32**	**7 (89)**	**3**	**1**	**0**
	34	0 (2)	1	2	1
	1015	0 (1)	2	2	0
	2148	1 (3)	1	2	1
	33	0 (1)	3	1	0
3	*3756*	0 (1)	0	1	14
	*3492*	4 (28)	1	6	8
	43	0 (2)	3	5	7
	44	0 (4)	1	5	9
	46	0 (1)	1	2	12
	**206**	**1 (9)**	**4**	**4**	**7**
	*3509*	0 (1)	1	4	10
	41	1 (3)	3	4	8
	2266	0 (5)	2	1	12
	2320	0 (1)	0	1	14
	1423	0 (1)	0	1	14
	*3502*	1 (1)	1	4	10
	*3508*	0 (1)	4	4	7
	1328	0 (2)	0	1	14
	2843	0 (1)	1	4	10
	40	0 (3)	2	1	12
4	1314	0 (11)	0	1	0
	*3757*	0 (1)	0	1	0
5	467	0 (1)	0	1	0
	13	0 (1)	0	1	0
6	**275**	**1 (4)**	**3**	**1**	**1**
	1163	0 (1)	2	1	2
	1671	0 (1)	1	2	2
	*3758*	0 (1)	0	1	4
	352	0 (1)	2	2	1
	*3435*	2 (3)	0	1	4
7	1154	0 (1)	1	0	0
	**60**	**0 (7)**	**1**	**0**	**0**
8	*3710*	0 (2)	0	2	1
	1323	0 (9)	1	2	0
	*3464*	0 (3)	1	0	2
	334	0 (1)	0	2	1
9	*3501*	0 (1)	1	0	0
	2986	0 (1)	1	0	0

*CC, clonal complex; ST, sequence type; freq, frequency of isolates with the ST; SLV, single locus variant; DLV, double locus variant; SAT, satellite. †We identified 9 clonal complexes and 17 singletons (11, 286, *5119*, 162, 1, *3759*, 23, 1943, 22, 944, *3471*, *3333*, 1011, 785, *3334*, *4178*, and *4013*), defined as strains that do not fit in any group in the collection. Some groups have an ST that is the ancestral strain for that group (shown in **boldface**). Unique STs are shown in *italics*.

Routine vaccination was initiated among children and young adults (<18 years of age) in late 2002 with a conjugated meningococcal vaccine against serogroup C (NeisVac-C, Baxter, Orth/Donau, Austria); >90% of all Icelanders <18 years of age were vaccinated. Three years later, no evidence has been seen for capsule switching.

### Antimicrobial Drug Susceptibility

All meningococcal isolates were susceptible to penicillin (MIC 0.012–0.125 μg/mL). All strains were also susceptible to rifampin (MIC 0.008–0.19 μg/mL) (*9*). In contrast, 148 isolates (40.9%) were resistant to sulfadiazine (MIC >8 μg/mL). Most commonly, these meningococci were ST-32, ST-1, and ST-11.

### Patient Outcomes

During the 28-year study period, 31 (8.6%) of 362 patients died after the infection. Higher case-fatality ratios were associated with higher age (p = 0.001), but no significant difference was seen between men and women (p = 0.953), residents in the capitol area and rural areas (p = 0.259), or patients who received treatment in hospitals in the capitol area versus in rural hospitals (p = 0.239). When results were analyzed by source of culture, patients with a positive culture from CSF only had significantly lower death ratios than other patients in the cohort (4.2% vs 11.5%, p = 0.02). These patients were younger than the remainder of the cohort, and as a result, this difference was of borderline significance when age was corrected for (p = 0.059).

The association between the most common STs, serogroups, and patient outcomes is summarized in Table 3. Unique STs were more frequently found in isolates with serogroup C capsule (19 of 204 with B, 33 of 144 with C, and 1 of 13 with other serogroups; p = 0.001). Death was not associated with particular serogroups, however (14 of 204 with B, 14 of 144 with C, and 3 of 14 with other serogroups, p = 0.138). The case-fatality ratio among patients infected by meningococci with previously described STs was 7.1% (22/309) compared to 17.0% (9/53) of patients infected by unique STs (p = 0.03). No significant difference was found between the age of patients with unique and previously described STs (p = 0.686) and source of isolates among patients with unique and previously described STs (p = 0.511).

We then performed multivariable logistic regression analysis to study the association of these parameters with outcome, age and sex of the patient, residence, location of hospital, source of positive culture, serogroup of the isolates, and ST group (unique STs in comparison to other STs). In the final model (Table 5) 3 parameters were independently associated with outcomes. Higher age was highly significantly associated with death, followed by infection with a unique ST. Isolation of meningococci from CSF only was associated with lower case-fatality ratios. Age and ST group remained significant when epidemic cases from 1977 were excluded from the analysis (data not shown).

**Table 5:** Multivariate analysis of death rate in patients with invasive meningococcal disease, Iceland, 1977–2004*

Variable	OR (95% CI)	p value
Age	1.031 (1.016–1.048)	0.0001
Unique ST†	3.225 (1.311–7.934)	0.011
Positive CSF culture‡	0.381 (0.147–0.984)	0.046

*OR, odds ratio; CI, confidence interval; ST, sequence type; CSF, cerebrospinal fluid. †Infection with an isolate with an ST unique to the country, in comparison to other, previously described STs. ‡Positive CSF culture only, in comparison to all other sources of positive culture, including blood only and blood and CSF.

## Discussion

To our knowledge, this population-based, longitudinal study is the first of its kind to examine the molecular epidemiology of all viable invasive meningococcal strains by using MLST. The 28-year observation period started in 1977, during an epidemic of meningococcal disease in Iceland. The well-defined population of Iceland, with excellent follow-up information on patients and its relative isolation make it an ideal setting for studies of this nature.

In the current study, the 362 isolates had 59 different STs, and of those, 19 were exclusively found in Iceland. These unique STs accounted for 14.4% of all infections during the 28-year period, and ST-3492 was by far the most common.

Although both long-term studies and population-based studies are lacking, other investigators have used MLST to study selected meningococcal strains from individual countries (*8**,**11**–**13*). For example, Murphy et al. analyzed 56 Irish meningococcal strains by this method, collected during a 4-year period. Of the invasive isolates, 26 different STs were identified, including 5 new ones (*12*). Takahasi et al. found 65 different STs among 182 isolates, 42 of which were unique to Japan, in a survey of Japanese strains (*13*). The distribution of some STs therefore seems to be fairly restricted geographically, which is also manifested by the fact that 41.1% of the Icelandic isolates characterized in this study are exclusively associated with Scandinavia.

ST-32 was the most common type found in Iceland, causing almost one fourth of all infections. It has been reported to cause numerous outbreaks worldwide and has a tendency to cause hyperendemic disease, particularly septicemia with a high death rate (*12*). However, in our study, the case-fatality ratio in patients infected by ST-32 did not differ from that in patients infected by other STs. The second most common type, ST-11, caused most cases of serogroup C disease during the second half of the study. This type has also been reported in several countries, with a propensity to spread rapidly once introduced into the population (*8**,**12**,**14*). The third most common type, ST-10, was mostly serogroup B. It was the primary cause of the meningococcal outbreak in 1977, but it disappeared after 1983. The most closely related type, ST-8, was first detected in the country more than a decade later, but this ST uniformly belonged to serogroup C. ST-3492 was the fourth most common type; it had not been described previously. It was almost uniformly serogroup C, with a tendency to cause worse outcomes.

By comparing the 7 housekeeping genes used in MLST, a close relationship between ST-11, ST-8, and ST-10 was observed. However, ST-11 and ST-8 are primarily serogroup C, whereas ST-10 is primarily serogroup B. These results could indicate genetic transfer and possible capsular switch. Analysis of genetic relatedness also shows a close relationship between ST-206 and ST-3492, which were most commonly serogroup C. However, both ST-206 and ST-3492 are part of the ST41/44 complex, which is predominantly associated with serogroup B meningococci, thus highlighting genetic transfer between closely related STs.

All of our strains were susceptible to penicillin. This contrasts with the situation in southern Europe, in particular, where resistance is increasingly reported (*15**–**17*). Likewise, none of the isolates in our study exhibited resistance to rifampin, which still seems to be rare (*18*).

To our knowledge, this is the first study to look at associations between STs and patient outcomes. By multivariable analysis, age and infection by a unique ST were independently associated with higher death rates. Lower death rates were observed among patients with a positive culture from CSF only than among other patients. Age has previously been shown to be associated with worse outcome in patients with meningitis (*19*), but infection with a novel or unique ST has not. At least 2 potential explanations could explain this difference. First, these strains likely represent evolutionary changes within the meningococcal population; therefore, a lower level of immunity against the unique STs within the population could translate into greater disease severity. In the case of pneumococcal infections, for example, the spread of clonal types can be influenced by herd immunity (*20*). Second, the difference in outcomes may indicate greater virulence of unique STs. Although data on this topic are lacking for meningococci, Sandgren et al. have shown that pneumococci with identical serotypes but different clonal types can have different invasive potentials (*21**,**22*). We therefore propose that meningococcal expression of virulence traits, other than the capsule type, may be linked to certain STs. Indeed, recent data suggest that serogroup C capsule expression may contribute to the invasive character of ST-11 meningococci (*23*). A more detailed analysis of virulence properties of specific meningococcal STs, including capsule expression, and their association with clinical characteristics is warranted. Judging from clinical experience, increased awareness during meningococcal epidemics may speed diagnosis and improve prognosis, which could bias our results since an epidemic of meningococcal infections was ongoing in 1977, when this study began. The epidemic was primarily caused by ST-10, an "old" ST, which accordingly could be associated with lower death ratio. However, the 2 risk factors for poor outcome remained significant even when epidemic cases were excluded, which argues against this hypothesis. When the outcomes were analyzed by source of the isolate, having a positive culture from CSF only was associated with lower risk for death. Although patients with CSF isolates were younger, this parameter remained significant when we corrected for age and ST category of the isolate. We do not have detailed information regarding patients' clinical signs and symptoms. Nevertheless, this part of the cohort most likely represents patients with meningitis, who generally have lower death ratios than do those with sepsis.

One limitation of the study is that submission date of MLST data ultimately determined whether we classified STs as old or new, which may bias the results. However, most data were submitted within a relatively short period, which should minimize this risk. As a result, more than a year from the original submission of the data (December 2005), we checked whether subsequent isolates with these novel STs had been identified, and none were found. Since routine vaccination was implemented in Iceland, meningococcal C disease has only been seen among unvaccinated adults. The rise in serogroup B is of concern, but a longer observation period is required before a conclusion can be reached regarding the issue of serogroup replacement.

In summary, this long-term, nationwide study looked at evolutionary trends of invasive meningococcal isolates in a well-defined setting, where invasive meningococcal disease has been hyperendemic. Although the most common STs have been described previously, we describe a high number of emerging STs. In particular, one ST, unique to Iceland, ranked fourth in prevalence. This study highlights the interplay between epidemiologic and evolutionary processes, which ultimately may produce unique meningococcal strains that lead to worse outcomes. More studies on virulence properties and host immunity are warranted to advance preventive strategies against meningococcal disease.
